# Sympatric *Ixodes*-tick species: pattern of distribution and pathogen transmission within wild rodent populations

**DOI:** 10.1038/s41598-018-35031-0

**Published:** 2018-11-09

**Authors:** Claire Cayol, Anu Jääskeläinen, Esa Koskela, Sami Kyröläinen, Tapio Mappes, Anja Siukkola, Eva R. Kallio

**Affiliations:** 1 University of Jyväskylä, Department of Biological and Environmental Science, P.O. Box 35, FI-40014 Jyväskylä, Finland; 2 University of Helsinki, Department of Virology, P.O. Box 21, FI-00014 Helsinki, Finland; 3 Helsinki University Central Hospital Laboratory Services (HUSLAB), Department of Virology and Immunology, P.O. Box 720, FI-00029 Helsinki, Finland; 4 University of Oulu, Department of Ecology and Genetics, PO Box 3000, FI-90014 Oulu, Finland

## Abstract

The generalist tick *Ixodes ricinus* is the most important vector for tick-borne pathogens (TBP), including *Borrelia burgdorferi sensu lato*, in Europe. However, the involvement of other sympatric *Ixodes* ticks, such as the specialist vole tick *I*. *trianguliceps*, in the enzootic circulations of TBP remains unclear. We studied the distribution of *I*. *ricinus* and *I*. *trianguliceps* in Central Finland and estimated the TBP infection likelihood in the most common rodent host in relation with the abundance of the two tick species. *Ixodes trianguliceps* was encountered in all 16 study sites whereas *I*. *ricinus* was frequently observed only at a quarter of the study sites. The abundance of *I*. *ricinus* was positively associated with open water coverage and human population density around the study sites. *Borrelia burgdorferi s*. *l*.-infected rodents were found only in sites where *I*. *ricinus* was abundant, whereas the occurrence of other TBP was independent of *I*. *ricinus* presence. These results suggest that *I*. *trianguliceps* is not sufficient, at least alone, in maintaining the circulation of *B*. *burgdorferi s*. *l*. in wild hosts. In addition, anthropogenic factors might affect the distribution of *I*. *ricinus* ticks and, hence, their pathogens, thus shaping the landscape of tick-borne disease risk for humans.

## Introduction

*Ixodes ricinus* is recognised as the most important vector for zoonotic tick-borne pathogens across Europe^1–4^. The European range of *I*. *ricinus* stretches from the British Isles to the Urals and from northern Scandinavia to Southern Europe^4,5^. However, the distribution of *I*. *ricinus* in nature is highly scattered due to its dependence on abiotic conditions and its sensitivity to desiccation^6–9^. The landscape structure, which determines the availability of appropriate host species, also contributes to the spatial distribution of *I*. *ricinus*^10–13^. Predicting the occurrence of *I*. *ricinus* remains challenging, but it is crucial for identifying the hazard represented by tick-borne pathogens. In addition, human exposure to ticks depends on the land use^14,15^. For instance, urban parks and forests involve a risk of exposure to the tick hazard for the growing urban human population during outdoor leisure activities^1,16^. The characterisation of favourable habitats for *Ixodes* species within these urban areas is, therefore, a critical step in the understanding of tick-borne diseases.

*Ixodes ricinus* transmits many pathogens of public health importance, including *Borrelia burgdorferi sensu lato* (*s*. *l*.), *Anaplasma phagocytophilum*, the tick-borne encephalitis virus (TBEV) and *Babesia* spp., responsible for Lyme borreliosis, granulocytic anaplasmosis, tick-borne encephalitis and babesiosis, respectively^1,17,18^. Meanwhile, other tick species may contribute to the circulation of these pathogens among wild vertebrate host species by increasing the infection prevalence in the wild hosts^1,19^. These enzootic transmission cycles need to be characterised to estimate and predict the risks that tick-borne zoonotic pathogens pose to humans. For instance, wild rodents are important reservoir hosts for many tick-borne pathogens^20–23^ as well as feeding hosts for *Ixodes* ticks. Of the *Ixodes* species that feed on rodents, *I*. *ricinus*, *I*. *trianguliceps* and *I*. *persulcatus* are found in Finland^24,25^. While *I*. *ricinus* and *I*. *persulcatus* are generalist ticks that bite a great variety of host species, including humans^26,27^, *I*. *trianguliceps* is a specialised nest-dwelling species that infests only small mammal hosts and hence does not pose a direct risk to humans^28^. Nevertheless, *I*. *trianguliceps* may contribute to the enzootic circulation of tick-borne pathogens among small mammals and, consequently, may indirectly be of medical and veterinary importance^1,19^.

Recently, the role of *I*. *trianguliceps* in circulating tick-borne pathogens among hosts has gained increasing attention. For instance, the transmission of certain *A*. *phagocytophilum* and *Babesia microti* strains has been shown to depend on *I*. *trianguliceps* rather than the coexisting *I*. *ricinus*^23^. In addition, it has been suggested that *I*. *trianguliceps* is a vector for TBEV^19,29^, *Candidatus* Rickettsia uralica^30^ and *Candidatus* Neoehrlichia mikurensis^31^. Moreover, *B*. *burgdorferi s*. *l*. has been detected in *I*. *trianguliceps* ticks that have been removed from small mammals, suggesting that *I*. *trianguliceps* may contribute to the transmission of this pathogen as well^32,33^. However, recent studies have suggested that the role of *I*. *trianguliceps* in the transmission of *B*. *burgdorferi s*. *l*. is less prominent than that of the coexisting *I*. *persulcatus*, due to its lower abundance and infection prevalence^33,34^. In Europe, *I*. *ricinus* has been proposed to be the primary vector for *B*. *burgdorferi s*. *l*., but the role of *I*. *trianguliceps* in its natural circulation among rodent host populations remains unclear.

Here, we analyse the distribution of *I*. *ricinus* and *I*. *trianguliceps* in nature, at the northernmost edge of its European geographical range, by examining questing ticks from the vegetation and tick infestation on rodents in both urban and non-urban forests. Moreover, we provide epidemiologically relevant data to clarify the role of *I*. *trianguliceps* in the circulation of *B*. *burgdorferi s*. *l*. in rodent populations. We hypothesise that *I*. *trianguliceps*, which is widely distributed in Fennoscandia^25,35,36^, alone supports the enzootic circulation of rodent-associated *B*. *burgdorferi s*. *l*. (*i*.*e*. *B*. *afzelii*)^37^ in locations where *I*. *ricinus* or *I*. *persulcatus* does not exist. To test this hypothesis, we examined *B*. *burgdorferi s*. *l*. infection prevalence in the most common rodent species in our study area, the bank vole (*Myodes glareolus*), in relation to *I*. *ricinus* and *I*. *trianguliceps* infestation at 16 independent study sites. For comparison, we also examined the bank voles for *A*. *phagocytophilum* and *B*. *microti*. The same strains of *B*. *microti* and *A*. *phagocytophilum* found from Finnish bank voles^38^ have been hypothesized to be circulated by *I*. *trianguliceps* ticks among the rodents in the UK^23,39,40^.

## Methods

### Ethical statement

The trapping and handling of wild bank voles was carried out in accordance with the Finnish Act on the Use of Animals for Experimental Purposes (62/2006). The methods applied on wild bank voles were approved by the Finnish Animal Experiment Board and the Finnish Ministry of the Environment, under the authorisation ESAVI/3834/04.10.03/2011.

### Data collection

The datasets analysed during the current study are available in the institutional repository of the University of Jyväskylä, http://urn.fi/URN:NBN:fi:jyu-201810094390.

The fieldwork was carried out at 16 study sites in and around Jyväskylä city area in Central Finland (see Supplementary Fig. S1, details in^36,41^). Eight of the study sites (sites 1, 2, 5, 6, 9, 10, 13 and 14) were located near settlement (‘urban sites’), while the other eight sites (sites 3, 4, 7, 8, 11, 12, 15 and 16) were located farther away from settlement (‘non-urban sites’) (see Supplementary Table S1). The study sites were examined with monthly rodent trappings and tick collections between May and September 2012. Rodent populations show regular three-year density cycles in the area^42^, and bank vole abundance was expected to be low in 2012. Therefore, to promote vole abundance, we provided approximately 8 litres of sunflower seeds once a month in half of the study sites after each trapping session^43^ (Supplementary Table S1). All study sites were situated in forest habitats dominated by spruce (*Picea abies*), pine (*Pinus sylvestris*) or mixed forests with spruce, pine and/or birch (*Betula* sp.), see also^41^. The sites were chosen to represent favourable habitats for the bank vole.

### Open water coverage and human population density in each study area

We assessed the inland open water coverage (in ha) or “*open water coverage*” around the trapping area (including lakes, ponds and rivers), in a circular area of 1 km radius (3.14 km^2^) centred on each trapping area, using GPS coordinates in Google Earth Pro. The 1 km radius area ensured that the entire sampling area was included (see below). We assessed the human population density “*human density*” within the same 3.14 km^2^ circular area, using LandScan, an accurate estimate of population density up to a resolution of 1 arc sec.^44^. The LandScanTM dataset was accessed and exploited on the online interface: https://www.populationexplorer.com/.

### Vole trapping

Small mammal trapping was carried out five times with *ca*. 4-week interval between May and September. At each study site, 20 Ugglan Special multi-capture live traps (Grahnab, Hillerstorp, Sweden) were set in two transects with ten traps in each transect with 10–15 m spacing between each trap. Before each trapping session, the traps were prebaited with sunflower seeds (*Helianthus annuus*) for 2–6 days, after which they were set for two consecutive days and nights and checked daily. The traps were removed from the field after each trapping session.

Captured bank voles were taken to a laboratory, where they were handled. When captured for the first time, each bank vole was individually marked with a microchip (Trovan Unique™) and an ear biopsy was taken. At each capture, but no more than once per trapping session (=month the sex and body mass of each individual was recorded and a blood sample (<200 μl) was taken from the retro-orbital sinus with capillary tubes (Haematocrit capillaries, Hirschmann Laborgeräte, Germany). Ear biopsy and blood samples were stored at ≤−20 °C until further examination. Upon each capture, the bank voles were also examined for ticks in bright light on the day of capture, with special attention paid to ears and the snout region (Supplementary Table S2). All ticks were removed and stored in 70% ethanol at −20 °C until later identification (see below). Male, juvenile and lactating female bank voles were released at the capture point immediately after handling. For the purpose of another research question (data not shown), obviously gravid females were kept in the laboratory until they gave birth, after which the litter quality and size were recorded (details of the methods in Koskela *et al*.^45^). Within a couple of days after giving birth, the mothers were released back to the field together with their offspring as described earlier^46^.

### Tick collection

Ticks were collected around the vole trapping transects during the week of rodents trapping^41^. Tick collection was carried out by dragging a 1 m^2^ (100 cm × 100 cm) cotton flannel in the vegetation for 300–500 meters per each vole trapping transect (600–1000 m per study site per session). The flag dragging was carried out within 100 m of the vole trapping transects. The flags were checked for ticks every 30–50 m, and the collected ticks were stored in 70% ethanol. The flag dragging was carried out predominantly between 9 am and 4 pm and only during dry weather. The tick flagging was not carried out during session 3 (July–early August) at four sites (sites 13–16), due to logistical issues.

### Tick identification

All ticks (removed from rodents and collected from vegetation) were identified based on morphological characteristics under a stereo and a light microscope using morphological identification keys^47–49^. Ticks were found to be either *I*. *ricinus* or *I*. *trianguliceps*; *I*. *persulcatus* was not detected. To further confirm the species identification, ten ticks that were morphologically identified as *I*. *ricinus* (7 individuals) and *I*. *trianguliceps* (3 individuals) were examined with molecular methods. DNA was extracted from ticks using alkaline digestion method^40^, followed by a PCR assay published by Caporale *et al*.^50^. The amplicons were successfully sequenced for eight samples. They confirmed our morphological tick identification^41^.

### Pathogen detection from voles

Pathogens were detected from the bank voles using ear biopsy (*B*. *burgdorferi s*. *l*.) or whole blood samples (*A*. *phagocytophilum* and *B*. *microti*) with PCR-based assays. Total DNA was extracted from the ear biopsy using a method described by Laird *et al*.^51^, whereas DNA was extracted from the blood using alkaline digestion^40^. DNA from blood samples was diluted 1:50 in sterile molecular-grade water to increase the quality of the signal and minimize the effect of DNA inhibitors that may be in the blood. DNA extracted from the ear biopsy was not diluted. Blank negative controls (one per every four samples) were subjected to all steps from extraction to PCR along with the samples. Moreover, one negative control consisting of molecular grade water was included in each real-time PCR plate. In the case of a negative control being positive, all samples around the contamination were rerun.

Real-time PCR methods were used for the detection of *A*. *phagocytophilum*^52^ and *B*. *microti*^23^ with small modifications. Briefly, for both assays the real-time PCR mix consisted of 0.5 µl of probe (10 pmol/µl), 22.5 pmol of each primer (10 pmol/µl), 12.5% of Itaq universal Probes Supermix, 2 µl of bovine serum albumin (5 mg/ml) and 2 µl of template, made up to a final volume of 20 µl with sterile molecular grade water. Cycling conditions in the BioRad CFX96 instrument were 5 min denaturation at 95 °C, followed by 50 cycles for *A*. *phagocytophilum* and 45 cycles for *B*. *microti* of 95 °C for 10 s and 60 °C for 1 min. The detection of *B*. *burgdorferi s*. *l*. was based on a published nested PCR assay targeting the *flaB* gene^53^. Since *B*. *burgdorferi s*. *l*. and *B*. *microti* cause chronic infections in rodents^54,55^, the probability of infection with each of the pathogens (see below) was estimated using only the first capture samples.

In total 317 blood samples from 247 bank voles, collected between May and August, were screened for the presence of anti-TBE virus antibodies using an immunofluorescence assay^56^. As none of the samples were positive (see Results), these data are not included in any further statistical analyses.

### Statistical analysis

The abundance of *I*. *ricinus* in vegetation was estimated as the sum of nymphs and adults flagged in vegetation at each site and during each session. Tick abundance was modelled in a generalised linear mixed model (GLMM) with zero-inflated negative binomial distribution and log link function. The fixed effects structure of the model included: the *session*, the *bank vole abundance* (defined as the total number of individuals trapped per site and per session), the total *open water coverage* (defined as a categorical variable with three levels: low, medium and high, based on the first (4.39 ha) and third (68.15 ha) quartiles of the measured water coverage in the area) and the *human density* in the area. Since the urban categorisation was significantly explained by the human population density (z = 6.66, p > 0.001), the two variables were not included in the same model. The interaction between human density and open water coverage was included in the full model. An offset term (log (distance flagged)/100) was introduced in the models to account for variation in the area flagged.

At the vole population level, the total number of immature ticks (larvae and nymphs) infesting bank voles per site and per session was modelled separately for both tick species, in a GLMM with negative binomial distribution and a log link function. The fixed effect structure of the model included *trapping session*, *open water coverage* categorised as described above and *human density* in the area, as well as their two-way interaction. The *log*(*trapped bank vole abundance*) was introduced as an offset in the model.

Tick infestation probability of bank voles (individual-level, binary response variable) was estimated separately for larvae and nymphs of both tick species (*I*. *trianguliceps* and *I*. *ricinus*) using the GLMM approach with binomial distribution and a logit link function. The individual-level explanatory variables assessed in each of the full models were *body mass* (centred value; used as a proxy for the age of the animal) and *body mass*^2^, *sex* and *body mass* * *sex* interaction term and *simultaneous infestation* (*yes/no*) *by the other life-stage of the same tick species* and *by the other tick species* (*i*.*e*. in the full model examining the probability of being infested with *I*. *trianguliceps* larvae, the explanatory variables for tick infestation were infestation by *I*. *trianguliceps* nymphs and infestation by any life stage of *I*. *ricinus*). The population-level explanatory variables in the full models were *location* (*i*.*e*. *urban/non-urban*) of the study site, provision of *supplementary food* (yes/no) and *trapping session* (categorical variable, 5 levels). Also, *I*. *ricinus abundance on the vegetation*, estimated as the sum of nymphs and adults collected per 100 m^2^ flag dragging per site during the entire study, was included in the full models to describe the overall abundance of *I*. *ricinus* at the site.

The infection probability of bank voles (individual level, binary response variable) at the first capture was estimated separately for *B*. *burgdorferi s*. *l*., *A*. *phagocytophilum* and *B*. *microti* using GLMMs with binomial distribution and a logit link function. The individual-level explanatory variables assessed in each of the full models were *body mass* (centred value), *body mass*^2^, *sex*, *sex* * *body mass* interaction term, *simultaneous tick infestation* (*yes/no*) *by I*. *trianguliceps*, *simultaneous tick infestation* (*yes/no*) *by I*. *ricinus* and *infection status for the other two pathogens*. The population-level explanatory variables in the full models were provision of *supplementary food* (yes/no), *trapping session* (categorical variable, 5 levels) and *I*. *ricinus abundance on the vegetation* (see above). The *location* (*i*.*e*. *urban/non-urban*) of the study site was not included in the full model due to convergence problems.

All models were fitted using the Laplace approximation method, using the lmer function in lme4 package or glmmadmb in glmmADMB package. Both packages were in R software^57^, available under GNU license at http://www.r-project.org. To control for the potential correlation amongst individuals that were captured at the same site, the site identity was included as a random effect in all models^58^. Due to a low mean number of captures per individual (mean 1.4), the identity of the individual was not taken into account as a random effect in the tick infestation models. Starting from the full models described above, a model selection was carried out based on corrected Akaike Information Criterion for small sample sizes (AICc) using ‘dredge’ function (library MuMIn) in R software. The simplest model within 2 AICc units of the model with the lowest AICc value was selected as the best model^59^ (see Supplementary Tables S3, S4 and S5).

## Results

### Tick abundance in vegetation

**Figure 1:**
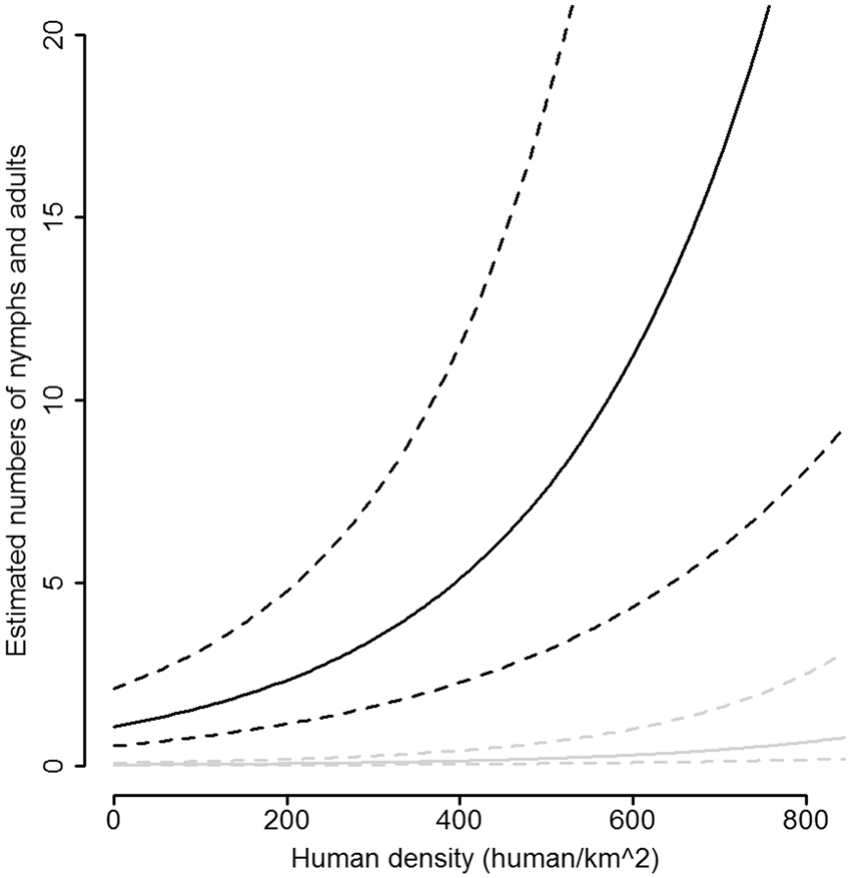
The estimated number (±SE) of nymphs and adults *I*. *ricinus* per 100 m^2^ of vegetation in relation to the open water coverage (low and medium water coverage in grey, high water coverage in black) and the human density (human/4 km^2^) observed in the study areas. The estimated values are assessed for the first session (May).

The total abundance of questing *I*. *ricinus* adults and nymphs showed temporal variation and was positively associated with total water coverage and human population density surrounding the study sites. The predicted abundance of questing ticks was *ca*. 0.5 in areas with low and medium water coverage and 23 in areas with high water coverage (68.15 to 199 ha) (Table 1 and Fig. 1). Within the range of observed human densities (0 to 845 human/km^2^) around our study sites, an increase in human population density of one unit (human/km^2^) increased tick abundance by 0.4% (Table 1 and Fig. 1).

**Table 1:** The abundance of *I*. *ricinus* nymphs and adults in vegetation (in log scale) in relation to explanatory variables selected for the best models.

		Coefficient (SE)	z-value	P-value
Intercept		0.071 (0.681)	0.10	0.917
Session	June	−0.530 (0.317)	−1.67	0.094
	July	−1.503 (0.355)	−4.24	<0.001
	August	−0.974 (0.325)	−2.99	0.003
	September	−1.545 (0.349)	−4.42	<0.001
Open water	Low	−3.636 (0.783)	−4.65	<0.001
	Medium	−3.798 (0.749)	−5.07	<0.001
Human density		0.004 (0.001)	3.57	<0.001
Random effect		σ^2^ = 0.681; sd = 0.825		

Session = trapping sessions (five levels, May is the reference), Open water = three levels: low, medium and high water coverage in the area (high is the reference), Human density = human population density in the area (human/km^2^). Random effect = identity of the study site. σ^2^ = the variance attributable to random effect. sd = standard deviation of σ^2^. The number of observations is 65.

### Tick infestation on bank voles

Each of the captured bank voles (n = 398) was examined for tick infestation during each capture (557 captures in total). *Ixodes trianguliceps* was found infesting voles in all 16 study sites (see Supplementary Table S1). *Ixodes ricinus* was observed in 11 sites and infesting voles in 10 sites, but all life-stages were frequently observed only in four sites (sites 1, 2, 5 and 6. see Supplementary Table S1). Even though the sites where *I*. *ricinus* was abundant were in close proximity to human settlement (*i*.*e*. *urban sites*), *I*. *ricinus* was not found at all *urban sites*.

Overall *I*. *trianguliceps* infestation prevalence on bank voles was 52.2%, with a mean infestation burden per infested vole of 3.30 ticks (±3.54 SD) (see Supplementary Table S2). Human population density in the area was not selected in the best model explaining *I*. *trianguliceps* infestation burden in the bank vole population, but open water coverage in the area was negatively correlated with the abundance of *I*. *trianguliceps* (Table 2). At the individual level, the probability of a bank vole being infested with *I*. *trianguliceps* larvae showed a nonlinear relationship with body mass, with the youngest individuals having the highest likelihood of being infested (Table 3). *Ixodes trianguliceps* nymph infestation increased the likelihood that a bank vole was infested with *I*. *trianguliceps* larvae. In addition, the infestation likelihood showed clear temporal variation, with the highest infestation likelihood in June and September and lowest in May and August. The likelihood that a bank vole was infested with *I*. *trianguliceps* nymphs (Table 3) was higher in male voles than in females, and simultaneous *I*. *trianguliceps* larval infestation increased the likelihood of nymphal infestation. *Ixodes trianguliceps* nymphal infestation probability was relatively stable over the summer but decreased significantly in autumn (September). At urban sites, the nymphal infestation likelihood was significantly lower than in non-urban sites (Table 3).

**Table 2:** Immature (larvae + nymphs) tick infestation burden on bank voles (in log scale) (a) *I*. *trianguliceps* and (b) *I*. *ricinus*, in relation to explanatory variables selected for the best models.

		(a) Immature *I*. *trianguliceps*			(b) Immature *I*. *ricinus*		
		Coefficient (SE)	z-value	P-value	Coefficient (SE)	z-value	P-value
Intercept		−0.750 (0.388)	−1.93	0.054	−0.143 (0.740)	−0.19	0.847
Session	June	0.461 (0.309)	1.49	0.136	1.001 (0.388)	2.58	0.010
	July	−0.538 (0.372)	−1.45	0.148	−0.651 (0.448)	−1.45	0.146
	August	0.133 (0.312)	0.43	0.669	−1.194 (0.437)	−2.74	0.006
	September	0.780 (0.314)	2.48	0.013	−0.543 (0.411)	−1.32	0.187
Open water	Low	0.981 (0.389)	2.52	0.012	−3.377 (0.841)	4.02	0.001
	Medium	0.914 (0.353)	2.59	0.010	−2.386 (0.701)	−3.41	0.001
Human density					0.002 (0.001)	2.21	0.027
Random effect		σ^2^ = 0.136; sd = 0.368			σ^2^ = 0.755; sd = 0.869		

Session = trapping sessions (five levels, May is the reference). Open water = total open water coverage in the area (ha); Random effect = identity of the study site. σ^2^ = The variance attributable to random effect. sd = Standard deviation of σ^2^. Offset = log (total number of voles trapped per site per session). The number of observations is 65.

**Table 3:** The probability of bank voles being infested with *I*. *trianguliceps* larvae and nymphs (in logit scale) in relation to explanatory variables selected for the best models.

		*I. trianguliceps* larva			*I. trianguliceps* nymph		
		Coefficient (SE)	z-value	P-value	Coefficient (SE)	z-value	P-value
Intercept		−1.596 (0.538)	−2.966	0.003	−0.800 (0.430)	−1.859	0.0630
BM		−0.187 (0.033)	−5.669	<0.001			
BM^2^		−0.014 (0.004)	−3.109	0.002			
Sex	Male				0.626 (0.215)	2.915	0.004
*I. tri*_L/N infestation		0.690 (0.252)	2.738	0.006	0.920 (0.243)	3.783	<0.001
Session	June	1.399 (0.558)	2.507	0.012	−0.133 (0.431)	−0.309	0.757
	July	0.141 (0.641)	0.220	0.826	−0.806 (0.487)	−1.654	0.098
	August	−0.539 (0.574)	−0.940	0.347	0.591 (0.413)	1.432	0.152
	September	1.566 (0.562)	2.785	0.005	−0.992 (0.447)	−2.221	0.026
Location	Urban				−0.797 (0.251)	−3.177	0.002
Random effect		σ^2^ = 0.016; sd = 0.126			σ^2^ = 0.059; sd = 0.242		

BM = the vole’s body mass (in grams, centred value), BM^2^ = polynomial term of BM; Session = trapping sessions (five levels, May is the reference); *I*. *tri_L/N* infestation = simultaneous *I*. *trianguliceps* larva/nymph infestation. Location = urban or non-urban location of the study site. Random effect = identity of the study site. σ^2^ = the variance attributable to random effect. sd = standard deviation of σ^2^. The number of observations is 551.

The overall infestation prevalence for *I*. *ricinus* across all study sites was 19.7% (Supplementary Table S2). The infestation prevalence was 30.9% among the voles captured from the 10 sites where *I*. *ricinus* was detected, and 56.6% in the four sites (sites 1, 2, 5 and 6) where *I*. *ricinus* was commonly found (see Supplementary Table S1). The mean *I*. *ricinus* infestation burden per infested bank vole was 3.03 (±3.94 SD) (Supplementary Table S2). The mean infestation burden with *I*. *ricinus* larvae and nymph in a given site per session was explained by the trapping session and by the total water coverage in the area, with larger mean infestation burdens in populations from areas with more open water (Table 2). The likelihood of an individual bank vole being infested with *I*. *ricinus* larvae or nymphs increased with age (body mass), and the likelihood was higher for males than for females (Table 4). In addition, the larval infestation likelihood varied during the study and was highest in early summer (May-June) and lowest in August. Both *I*. *ricinus* larval and nymphal infestation likelihoods were positively associated with the abundance of *I*. *ricinus* (nymphs and adults) collected from the vegetation (Table 4).

**Table 4:** The probability of bank vole being infested with *I*. *ricinus* larvae and nymphs (in logit scale) in relation to explanatory variables selected for the best models.

		*I. ricinus* larva			*I. ricinus* nymph		
		Coefficient (SE)	z-value	P-value	Coefficient (SE)	z-value	P-value
Intercept		−3.735 (0.818)	−4.564	<0.001	−7.369 (1.089)	−6.770	<0.001
BM		0.090 (0.032)	2.797	0.005	0.182 (0.062)	2.937	0.003
Sex	Male	1.137 (0.337)	3.372	<0.001	2.851 (0.932)	3.058	0.002
*I. ric*_L infestation					1.477 (0.670)	2.205	0.027
Session	June	0.632 (0.613)	1.030	0.303			
	July	−1.003 (0.613)	−1.501	0.133			
	August	−1.387 (0.646)	−2.148	0.032			
	September	−0.072 (0.644)	−0.111	0.912			
*I. ric* abundance		1.422 (0.362)	3.924	<0.001	0.594 (0.207)	2.863	0.004
Random effect		σ^2^ = 1.758; sd = 1.326			σ^2^ = 0; sd = 0		

BM = the vole’s body mass (in grams, centred value); Session = trapping sessions (5 levels); *I*. *ric* abundance = the abundance of *I*. *ricinus* (nymphs and adults together) on vegetation (per 100 m of flag dragging) in the study site over the study period; *I*. *ric_L* infestation = simultaneous *I*. *ricinus* larva infestation. Random effect = identity of the study site. σ^2^ = The variance attributable to random effect. sd = Standard deviation of σ^2^. The number of observations is 551.

### Pathogen infections in the bank vole

Only the first samples of wild-born bank voles were used to detect *B*. *burgdorferi s*. *l*., *A*. *phagocytophilum* and *B*. *microti* infections from bank voles. In total 39 (out of 349 first-capture ear tissue samples) bank voles were infected with *B*. *burgdorferi s*. *l*., all of which were captured from four sites (sites 1, 2, 5 and 6) where *I*. *ricinus* was commonly observed (Supplementary Table S1). At these four sites, *B*. *burgdorferi s*. *l*. infection prevalence varied between 28 and 47% in the first-capture samples (Supplementary Table S1). *Anaplasma phagocytophilum* and *B*. *microti* infections were examined from 329 first-capture blood samples, of which 114 and 156 were found positive, respectively. *Babesia microti-*infected bank voles were found in all 16 study sites, with a prevalence of 16–72% among the first-capture samples. *Anaplasma phagocytophilum*-infected bank voles were found in 15 sites, in which the prevalence varied between 17 and 75% among the first-capture samples (Supplementary Table S1).

**Figure 2:**
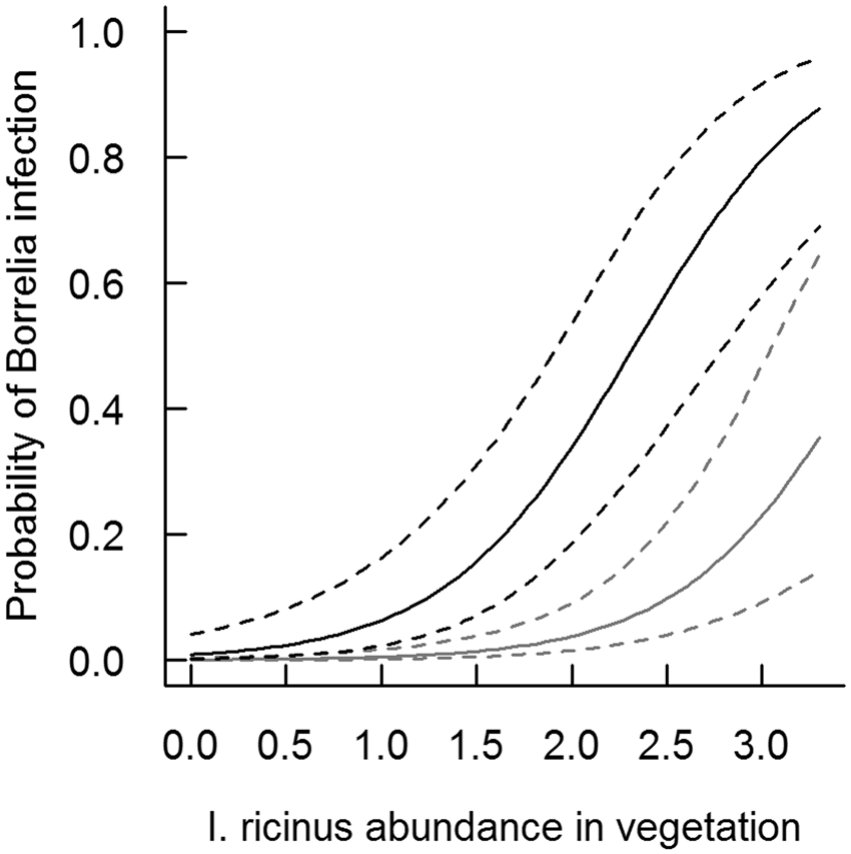
The predicted likelihood (±95% CI) of a bank vole (female in grey, male in black) being *B*. *burgdorferi s*. *l*. infected at the first capture in relation to the *I*. *ricinus* abundance per 100 m^2^ of vegetation in the study site. The predicted likelihood is assessed for an individual of average weight not infested with *I*. *trianguliceps*.

The probability of a bank vole being infected with *B*. *burgdorferi s*. *l*. (Table 5 and Fig. 2) at the first capture was higher for heavier (=older) individuals and for males. *B*. *burgdorferi s*. *l*. infection likelihood showed a significant positive association with the abundance of *I*. *ricinus* nymphs and adults observed at the site during the study (Table 5, Fig. 2). *Ixodes trianguliceps* infestation decreased the probability of infection with *B*. *burgdorferi s*. *l*. The probability of infection with *A*. *phagocytophilum* at the first capture was negatively associated with individual body mass (=age) and the observed abundance of *I*. *ricinus* at the site and positively associated with simultaneous *B*. *microti* infection (Table 5). *Babesia microti* infection likelihood, in turn, showed a non-linear relationship with the individual’s body mass and a positive relationship with simultaneous *A*. *phagocytophilum* infection (Table 5).

**Table 5:** The probability of infection in a bank vole with (a) *B*. *burgdorferi s*. *l*. (in logit scale), (b) *A*. *phagocytophilum* and (c) *B*. *microti* in relation to the explanatory variables selected to the best models.

	Coefficient (SE)	z-value	P-value
(**a**) ***B***. ***burgdorferi s***. ***l***.			
Intercept	−7.295 (1.700)	−4.292	<0.001
BM	0.186 (0.051)	3.615	<0.001
Sex Male	2.568 (0.687)	3.736	<0.001
IT	−1.228 (0.620)	−1.982	0.048
*I*. *ric* abundance	2.029 (0.659)	3.079	0.002
Random effect	σ^2^ = 2.097; sd = 1.448		
(**b**) ***A***. ***phagocytophilum***			
Intercept	−0.891 (0.273)	−3.261	0.001
BM	−0.076 (0.029)	−2.656	0.008
*B*. *microti* infection	0.761 (0.280)	2.718	0.007
*I*. *ric* abundance	−0.415 (0.190)	−2.186	0.029
Random effect	σ^2^ = 0.291; sd = 0.540		
(**c**) ***B***. ***microti***			
Intercept	0.040 (0.213)	0.189	0.850
BM	0.125 (0.030)	4.211	<0.001
BM^2^	−0.017 (0.004)	−3.972	<0.001
*A*. *phagocytophilum* infection	0.841 (0.285)	2.953	0.003
Random effect	σ^2^ = 0.160; sd = 0.399		

BM = body mass (in grams, centred value); IT = simultaneous infestation by *I*. *trianguliceps* larvae, nymphs or both; *I*. *ric* abundance = the abundance of *I*. *ricinus* (nymphs and adults together) on vegetation (per 100 m of flag dragging) in the study site over the study period. Random effect = identity of the study site. σ^2^ = The variance attributable to random effect. sd = Standard deviation of σ^2^. The number of observations is 304.

In total, 317 blood samples (247 first capture, 70 subsequent samples) were screened for anti-TBEV antibodies. One sample was suspected to be positive, but a subsequent sample taken one month later from the same individual was negative, and the individual was concluded to be uninfected. Consequently, there were no indications that TBEV circulated in bank voles at our study areas in 2012.

## Discussion

In this study, we analysed factors affecting the local distribution of *I*. *ricinus* at the northernmost part of its geographical range. We found that free-living and parasitic stages of *I*. *ricinus* were more likely to be present at the sites with the largest open water coverage and highest human density. We examined the hypothesis that *I*. *trianguliceps*, another rodent-specific tick species, has a crucial role in the epidemiology of *B*. *burgdorferi s*. *l*. independent of the presence of *I*. *ricinus* (or *I*. *persulcatus*). Our data do not support this hypothesis: the infection likelihood with *B*. *burgdorferi s*. *l*. in bank voles showed a negative rather than a positive relationship with *I*. *trianguliceps* infestation. In addition, *B*. *burgdorferi s*. *l*. infection did not show any relationship (*i*.*e*. not selected into the best model) with *A*. *phagocytophilum* or *B*. *microti* infections, certain rodent strains of which have been found to be associated with *I*. *trianguliceps*^23,38–40^. Instead, the role of *I*. *ricinus* as the vector of *B*. *burgdorferi s*. *l*. was highlighted: infected voles were found only from the sites where *I*. *ricinus* was frequently observed. Moreover, the bank voles’ likelihood of being infected with *B*. *burgdorferi s*. *l*. was positively related to the observed abundance of *I*. *ricinus* in the vegetation. These results further suggest that *I*. *trianguliceps* does not play a major role in the transmission of *B*. *burgdorferi s*. *l*. among rodent hosts^32^. Another tick species such as *I*. *ricinus* is required to support the transmission and persistence of this pathogen among rodent populations^33,34^.

*A*. *phagocytophilum* and *B*. *microti* infection likelihoods did not show positive relationships either with *I*. *ricinus* abundance or simultaneous *B*. *burgdorferi s*. *l*. infection (Table 5). This suggests that *A*. *phagocytophilum* and *B*. *microti* are transmitted by a different vector than *B*. *burgdorferi s*. *l*. and that they are not dependent on *I*. *ricinus* for transmission. Previous studies^23,39,40^ have suggested that the rodent-associated strains of *A*. *phagocytophilum* and *B*. *microti* found in the UK, which have also been found in our study area in Finnish bank voles^38^, are circulated among voles by *I*. *trianguliceps*. We found *B*. *burgdorferi s*. *l*.-infected voles only in sites where *I*. *ricinus* was frequently observed, while *B*. *microti*- and *A*. *phagocytophilum*-infected animals were found in all sites. These findings further support the conclusion that the vector of *B*. *burgdorferi s*. *l*. in our study system is *I*. *ricinus*, while the role of *I*. *trianguliceps* is likely to be minor.

The observed negative relationship between *B*. *burgdorferi s*. *l*. infection and simultaneous *I*. *trianguliceps* infestation is likely to result from the difference in bank voles’ exposure to these parasites: *Ixodes trianguliceps* infestation decreased with age (body mass; Table 3) indicating that voles become exposed to the tick in early life, reflecting the nidicolous nest-dwelling lifestyle of the tick^28^. *Borrelia burgdorferi s*. *l*. infection likelihood, however, increases with age (Table 5), as does *I*. *ricinus* infestation likelihood (Table 4), reflecting the questing behaviour of the tick and further supporting the conclusion that *I*. *ricinus* is responsible for the transmission of *Borrelia* to bank voles in our study system. The lack of the expected positive relationship between *B*. *burgdorferi s*. *l*. infection likelihood and simultaneous *I*. *ricinus* infestation (*I*. *ricinus* infestation was not selected in the best model) may result from the time delay from infection (infestation by an infected tick) to detection from ear biopsy samples, which takes approximately one month^60,61^.

*Ixodes trianguliceps*, which spends the off-host phase of its life-cycle in host burrows, was found infesting voles in all study sites, while *I*. *ricinus* was frequently questing in the vegetation and infesting voles only in a quarter (4/16) of these sites. Although the sites where *I*. *ricinus* was frequently observed were all ‘*urban sites’* (*i*.*e*. located close to settlement), this tick species was not found in every site categorised as urban. Instead, we found that sites with larger open water coverage and higher human densities were more likely to show abundant *I*. *ricinus* populations, both on bank voles and in vegetation. Humidity is one of the main abiotic factors allowing the establishment of *I*. *ricinus*^6,62^, and areas with large open water coverage may offer favourable moisture conditions for *I*. *ricinus* to colonise. A similar relationship was found for *I*. *scapularis* in the USA and tick-borne diseases in Sweden^15,63^. However, negative or non-significant correlations between tick abundance and water coverage were also found, depending on the tick species and the methodology used^64–66^. Regarding urban sites, areas with dense human populations can have several anthropogenic characteristics that may promote *I*. *ricinus* populations. These include higher temperatures^67,68^, garden resource provisioning that benefits important tick hosts such as deer^69^, and lower species diversity, favouring ubiquitous species such as rodents^67,70^. It is important to note that other factors such as host species assemblage, soil characteristics, habitat connectivity and other landscape attributes could also contribute to the patchiness of *I*. *ricinus*^13^ and that these need to be addressed in future studies. Furthermore, the analysis of infection prevalence in questing *I*. *ricinus* could provide additional information. Nevertheless, we propose that urban areas provide favourable habitats to *I*. *ricinus* and may even serve as stepping stones for this species in its ongoing spread northwards.

In all, our study suggests that abiotic and anthropogenic factors influence the patchy nature of *I*. *ricinus* distribution and that sympatric and taxonomically closely related vector species can have different effects on the epidemiology of the pathogens they harbour. These results may help to assess human risk for tick-borne diseases, especially Lyme Borreliosis, in urban areas. These findings are especially important, considering the lower awareness and altered perception of tick-borne disease risk in dwellers of urban areas compared with those in rural settings^71,72^.

## Electronic supplementary material

Supplementary information

## Data Availability

The datasets analysed during the current study are available in the institutional repository of the University of Jyväskylä, http://urn.fi/URN:NBN:fi:jyu-201810094390.
