# Supplementary material for: Sympatric Ixodes-tick species: pattern of distribution and pathogen transmission within wild rodent populations
Source: Sci Rep. 2018 Nov 9;8:16660. doi: 10.1038/s41598-018-35031-0 (PMC6226450; doi:10.1038/s41598-018-35031-0)
Supplement: Supplementary file 1 — Supplementary information [file 41598_2018_35031_MOESM1_ESM.pdf]

Supplementary file

Sympatric *Ixodes*-tick species: pattern of distribution and pathogen transmission within wild rodent populations

Claire Cayol[1], Anu Jääskeläinen[2,3], Esa Koskela[1], Sami Kyröläinen[1], Tapio Mappes[1], Anja Siukkola[1] & Eva R. Kallio[1,4]

1. University of Jyväskylä, Department of Biological and Environmental Science, P.O. Box 35, FI-40014 University of Jyväskylä, Finland, claire.c.cayol@gmail.com; esa.m.koskela@jyu.fi; sami.t.kyrolainen@jyu.fi; anja.siukkola@gmail.com; tapio.mappes@jyu.fi

2. University of Helsinki, Department of Virology, P.O. Box 21, FI-00014 University of Helsinki, Finland, anu.jaaskelainen@helsinki.fi

3. Helsinki University Central Hospital Laboratory Services (HUSLAB), Department of Virology and Immunology, P.O. Box 720, FI-00029 HUS, Finland

4. University of Oulu, Department of Ecology and Genetics, PO Box 3000, 90014 Oulu, Finland, Eva.Kallio@oulu.fi

* Corresponding author: claire.c.cayol@gmail.com, +358 40 484 3717

Table S1. Summary of the data with the number of bank vole captures (BV), *I. trianguliceps* (*I. tri* %) and *I. ricinus* (*I. ric* %) infestation prevalence in bank voles, the mean abundance of *I. ricinus* (*I. ric*) per 100m^2 in vegetation and pathogen infection in bank voles at each of the 16 study sites over the study period (May-Sep 2012). U/N = Location of the site; urban (U) or non-urban location (N). Food = Food supplementation treatment (yes/no). B.b = *Borrelia burgdorferi s.l.*, A.p = *Anaplasma phagocytophilum*, B.mi = *Babesia microti* (infected/total tested when considering first captures). Sites with frequent observations of *I. ricinus* are in bold.

Site	U/N	Food	BV	*I.tri* %	*I.ric* %	*I.ric*	B.b	A.p	B.mi
1	U	No	28	39	46	1.13	5/18	6/17	11/17
2	U	Yes	35	46	60	5.73	11/28	8/28	12/28
3	N	Yes	58	50	3	0	0/39	6/36	14/36
4	N	No	15	47	0	0.1	0/7	2/7	3/7
5	U	Yes	27	26	52	1.70	6/21	4/17	9/17
6	U	No	55	36	62	3.49	17/36	0/31	5/31
7	N	No	24	67	8	0.04	0/18	5/16	9/16
8	N	Yes	38	50	16	0.02	0/22	5/20	9/20
9	U	No	33	52	18	0.09	0/25	12/25	18/25
10	U	Yes	51	63	22	0	0/27	14/25	12/25
11	N	Yes	10	60	0	0	0/4	3/4	1/4
12	N	No	7	71	14	0.04	0/6	2/6	3/6
13	U	Yes	45	56	0	0	0/24	14/24	15/24
14	U	No	63	56	0	0	0/37	13/36	16/36
15	N	No	16	63	0	0	0/6	4/6	2/6
16	N	Yes	52	69	0	0	0/31	16/31	17/31

Table S2. Ticks removed from the voles during the study

Tick species	Range	Ticks total	Infested voles*	% of voles infested*	Mean tick burden (SD)*	Mean tick burden infested vole (SD) ~
I. trianguliceps						
Larva	0-19	503	168	30.2	0.90 (2.33)	1.71 (3.01)
Nymph	0-12	388	186	33.4	0.70 (1.49)	1.33 (1.84)
Female	0-6	67	36	6.5	0.12 (0.56)	0.23 (0.76)
Male	0-2	3	2	0.4	0.01 (0.10)	0.01 (0.13)
Total	0-21	961	291	52.2	1.73 (3.04)	3.30 (3.54)
I. ricinus						
Larva	0-27	301	105	18.9	0.54 (1.94)	2.74 (3.62)
Nymph	0-4	31	20	3.6	0.06 (0.34)	0.28 (0.72)
Female	0-1	1	1	0.2	0.00 (0.04)	0.01 (0.10)
Total	0-29	333	110	19.7	0.60 (2.12)	3.02 (3.94)

* 557 vole observations, *i.e.* all captured bank voles.

~ 291 vole observations for *I. trianguliceps*, 110 for *I. ricinus*, *i.e.* only individuals infested by the given tick species.

Table S3. Model selection table for models showed in tables 1 and 2. Full model, best model, and all models laying at 2 AICc difference from the lowest AICc are showed with their degree of freedom (Df).

The explanatory variables included in the full models are Session, bank vole abundance (BV), human density in the area (Human density), open water coverage (Open Water), which is a categorical variable with 3 levels based on the first and third quartile of the measured open water coverage in the area (in ha), and relevant interactions.

Model for the abundance of *I. ricinus* nymphs+adults in the vegetation (Table 1)		Df	AICc	Delta
Full	Session + BV + Human density + Open water + Human density * Open water			
Best	Session + Human density + Open water	11	213.8	0.00
Other	Session + BV + human density + Open water	12	214.4	0.57
	BV + Human density + Open water	8	214.5	0.68
	Session + Human density + Open water + Human density * Open water	13	215.1	1.30
Model for the abundance of *I. trianguliceps* nymphs + larvae infesting voles (Table 2a)				
Full	Session + Human density + Open water + Human density * Open water			
Best	Session + Open water	9	417.3	0.00
Other	Human density + Session + Open water	8	418.3	1.01
	Human density + Session	8	418.3	1.06
	Session	7	418.4	1.17
Model for the abundance of *I. ricinus* nymphs + larvae infesting voles (Table 2b)				
Full	Session + Human density + Open water+ Human density * Open water			
Best	Session + Human density + Open water	10	258.0	0.00
Other	Session + Open water	9	259.2	1.25

Table S4. Model selection table for models showed in table 3 and 4. Full model, best model, and all models laying at 2 AICc difference from the lowest AICc are showed with their degree of freedom (Df).

The explanatory variables included in the full models are body mass (BM, centred value) and its second-degree polynomial term (BM^2), sex (Sex), simultaneous infestation with *I. trianguliceps* larvae or nymph (*I. tri*_L, and *I. tri*_N, yes/no), simultaneous infestation with *I. ricinus* larvae or nymph (*I. ric*_L, and *I. ric*_N, yes/no), presence of any stage *I. trianguliceps* or *I. ricinus* (IT, IR yes/no), location of the study site (urban, yes/no), provision of supplementary food (food, yes/no), trapping session (Session) and abundance of *I. ricinus* on the vegetation, estimated as the sum of nymphs and adults collected per 100 m^2 flag dragging per site during the entire study (*I. ric* abundance).

Model for the probability of an individual bank vole being infested with *I. trianguliceps* larvae (Table 3)		Df	AICc	Delta
Full	BM + BM^2 + Sex + BM * Sex + *I. tri*_N + IR + Session + Urban + *I. ric* abundance+ Food			
Best	BM + BM^2+ *I. tri*_N + Session	9	507.8	0.00
Other	BM + BM^2+ *I. tri*_N + Session + *I. ric* abundance	10	508.5	0.73
	BM + BM^2+ *I. tri*_N + Session + Sex	10	508.7	0.90
	BM + BM^2+ *I. tri*_N + Session + Urban + *I. ric* abundance	11	509.2	1.44
	BM + BM^2+ *I. tri*_N + Session + Sex + *I. ric* abundance	11	509.5	1.72
	BM + BM^2+ *I. tri*_N + Session + Urban	10	509.6	1.80
	BM + BM^2+ *I. tri*_N + Session + Food	10	509.6	1.86
	BM + BM^2+ *I. tri*_N + Session + IR	10	509.7	1.96
Model for the probability of an individual bank vole being infested with *I. trianguliceps* nymphs (Table 3)				
Full	BM + BM^2 + Sex + BM * Sex + *I. tri*_L + IR + Session + Urban + *I. ric* abundance+ Food			
Best	*I. tri*_L + Session + Sex + Urban	9	592.5	0.00
Other	*I. tri*_L + Session + Sex + Urban + BM^2	10	592.6	0.12
	*I. tri*_L + Session + Sex + Urban + IR	10	593.7	1.26
	*I. tri*_L + Session + Sex + Urban + BM^2 + IR	11	593.9	1.44
	*I. tri*_L + Session + Sex + Urban + BM + BM^2	11	593.9	1.46
	*I. tri*_L + Session + Sex + Urban + BM	10	594.2	1.69
	*I. tri*_L + Session + Sex + Urban + *I. ric* abundance	10	594.2	1.73
	*I. tri*_L + Session + Sex + Urban + Food	10	594.3	1.87
	*I. tri*_L + Session + Sex + Urban + BM^2+ *I. ric* abundance	11	594.4	1.91
Model for the probability of an individual bank vole being infested with *I. ricinus* larvae (Table 4)				
Full	BM + BM^2 + Sex + BM * Sex + IT + *I. ric*_N + Session + Urban + Food + *I. ric* abundance			
Best	BM + Sex + Session + *I. ric* abundance	9	351.1	0.80
Other	BM + Sex + Session + *I. ric* abundance+ *I. ric*_N + BM * Sex	11	350.3	0.00
	BM + Sex + Session + *I. ric* abundance+ BM * Sex	10	350.4	0.09

	BM + Sex + Session + *I. ric* abundance+ *I. ric*_N	10	350.5	0.19
	BM + Sex + Session + *I. ric* abundance+ Urban + BM * Sex	11	350.6	0.29
	BM + Sex + Session + *I. ric* abundance+ *I. ric*_N + Urban + BM * Sex	12	350.7	0.34
	BM + Sex + Session + *I. ric* abundance+ *I. ric*_N + Urban	11	350.9	0.59
	BM + Sex + Session + *I. ric* abundance+ Urban	10	351.4	1.06
	BM + Sex + Session + *I. ric* abundance+ *I. ric*_N + IT	11	351.6	1.26
	BM + Sex + Session + *I. ric* abundance+ IT + BM * Sex	11	351.6	1.28
	BM + Sex + Session + *I. ric* abundance+ *I. ric*_N + IT + BM * Sex	12	351.6	1.28
	BM + Sex + Session + *I. ric* abundance+ IT + Urban + BM * Sex	12	351.8	1.44
	BM + Sex + Session + *I. ric* abundance+ *I. ric*_N + IT + Urban + BM * Sex	13	351.9	1.59
	BM + Sex + Session + *I. ric* abundance+ *I. ric*_N + IT + Urban	12	352.0	1.62
	BM + BM^2 + Sex + Session + *I. ric* abundance+ *I. ric*_N	11	352.0	1.67
	BM + Sex + Session + *I. ric* abundance+ IT	10	352.1	1.72
	BM + Sex + Session + *I. ric* abundance+ IT + Urban	11	352.3	1.93
Model for the probability of an individual bank vole being infested with *I. ricinus* nymphs (Table 4)				
Full	BM + BM^2 + Sex + BM * Sex + IT + *I. ric*_L + Session + Urban + Food + *I. ric* abundance			
Best	BM + *I. ric*_L + Sex + *I. ric* abundance	6	120.0	1.60
Other	BM + BM^2 + *I. ric*_L + Sex+ *I. ric* abundance+ BM * Sex	8	118.4	0.00
	BM + BM^2 + *I. ric*_L + Sex+ *I. ric* abundance+ BM * Sex + Urban	9	118.5	0.09
	BM + BM^2 + *I. ric*_L + Sex+ *I. ric* abundance+ Food + BM * Sex	9	118.8	0.31
	BM + BM^2 + *I. ric*_L + Sex+ *I. ric* abundance	7	119.3	0.82
	BM + BM^2 + *I. ric*_L + Sex+ *I. ric* abundance+ Food + BM * Sex + Urban	10	119.3	0.90
	BM + BM^2 + *I. ric*_L + Sex+ *I. ric* abundance+ Food	8	119.4	1.00
	BM + BM^2 + *I. ric*_L + Sex+ *I. ric* abundance+ Urban	8	119.7	1.22
	BM + BM^2 + *I. ric*_L + Sex+ *I. ric* abundance+ Food + Urban	9	120.3	1.81
	BM + BM^2 + *I. ric*_L + Sex+ *I. ric* abundance+ IT + BM * Sex	9	120.3	1.91
	BM + BM^2 + *I. ric*_L + Sex+ *I. ric* abundance+ IT + BM * Sex + Urban	10	120.4	1.92

Table S5. Model selection table for models showed in table 5. Full model, best model, and all models laying at 2 AICc difference from the lowest AICc are showed with their degree of freedom (Df).

The explanatory variables included in the full models are *A. phagocytophilum* (A.p), *B. microti* (B.mi) and *B. burgdorferi s.l.* (B.b) infection, body mass (BM, centred value) and its second-degree polynomial term (BM^2), sex (Sex), infestation with larvae, nymphs of both stages of *I. trianguliceps* (IT, yes/no) and *I. ricinus* (IR, yes/no), the location of the study site (urban, yes/no), the provision of supplementary food (food, yes/no), trapping session (session), and the abundance of *I. ricinus* on the vegetation, estimated as the sum of nymphs and adults collected per 100 m^2 flag dragging per site during the entire study (*I. ric* abundance).

Model for the probability of an individual bank vole being infected with *B. burgdorferi s.l.* (Table 5)		Df	AICc	Delta
Full	A.p + B.mi + Sex + IT + IR + BM + BM^2 + BM * Sex + Session + Food + *I. ric* abundance			
Best	BM + IT + Sex + *I. ric* abundance	6	121.6	0.00
Other	BM + Sex + *I. ric* abundance+ BM^2 + IT	7	121.6	0.02
	BM + Sex + *I. ric* abundance+ BM^2 + A.p + IT	8	121.6	0.06
	BM + Sex + *I. ric* abundance+ A.p + IT	7	122.0	0.50
	BM + Sex + *I. ric* abundance+ B.mi + IT	7	122.3	0.72
	BM + Sex + *I. ric* abundance+ IR + IT	7	122.4	0.80
	BM + Sex + *I. ric* abundance+ BM^2	6	122.4	0.87
	BM + Sex + *I. ric* abundance+ BM^2 + A.p + IR + IT	9	122.5	0.90
	BM + Sex + *I. ric* abundance+ A.p + IT + BM * Sex	8	122.5	0.95
	BM + Sex + *I. ric* abundance+ IT + BM * Sex	7	122.6	1.06
	BM + Sex + *I. ric* abundance+ BM^2 + IR + IT	8	122.6	1.07
	BM + Sex + *I. ric* abundance+ A.p + IR + IT	8	122.6	1.08
	BM + Sex + *I. ric* abundance+ BM^2 + B.mi + IT	8	122.7	1.16
	BM + Sex + *I. ric* abundance+ BM^2 + A.p	7	122.9	1.30
	BM + Sex + *I. ric* abundance+ A.p + B.mi + IT	8	123.1	1.53
	BM + Sex + *I. ric* abundance+ BM^2 + A.p + B.mi + IT	9	123.1	1.56
	BM + Sex + *I. ric* abundance+ B.mi + IR + IT	8	123.1	1.59
Model for the probability of an individual bank vole being infected with *A. phagocytophilum* (Table 5)				
Full	B.b + B.mi + Sex + IT + IR + BM + BM^2 + BM * Sex + Session + Food + *I. ric* abundance			
Best	BM + B.mi + *I. ric* abundance	5	360.1	0.00
Other	BM + B.mi + *I. ric* abundance+ Food	6	360.6	0.49
	BM + B.mi + *I. ric* abundance+ B.b	6	361.2	1.04
	BM + B.mi + *I. ric* abundance+ B.b + Food	7	361.6	1.51
	BM + B.mi + *I. ric* abundance+ IR	6	361.7	1.55
	BM + B.mi + *I. ric* abundance+ IT	6	362.0	1.87

Model for the probability of an individual bank vole being infected with *B. microti* (Table 5)				
Full	B.b + A.p + Sex + IT + IR + BM + BM^2 + BM * Sex + Session + Food + *I. ric* abundance			
Best	BM + BM^2 + A.p	5	385.7	0.84
	BM + BM^2 + A.p + *I. ric* abundance	6	384.8	0.00
	BM + BM^2 + A.p + IT + *I. ric* abundance	7	385.0	0.20
	BM + BM^2 + A.p + IT	6	385.3	0.47
	BM + BM^2 + A.p + Food + *I. ric* abundance	7	385.4	0.56
	BM + BM^2 + A.p + Sex + *I. ric* abundance	7	385.6	0.82
	BM + BM^2 + A.p + IT + Food + *I. ric* abundance	8	385.7	0.86
	BM + BM^2 + A.p + IT + Sex + *I. ric* abundance	8	386.1	1.25
	BM + BM^2 + A.p + Sex + Food + *I. ric* abundance	8	386.2	1.40
	BM + BM^2 + A.p + IT + Sex	7	386.4	1.53
	BM + BM^2 + A.p + IT + Food	7	386.4	1.59
	BM + BM^2 + A.p + Sex	6	386.4	1.63
	BM + BM^2 + A.p + IT + Sex + Food + *I. ric* abundance	9	386.7	1.92
	BM + BM^2 + A.p + Food	6	386.7	1.92
	BM + BM^2 + A.p + IR + *I. ric* abundance	7	386.8	1.94
	BM + BM^2 + A.p + B.b + *I. ric* abundance	7	386.8	1.96

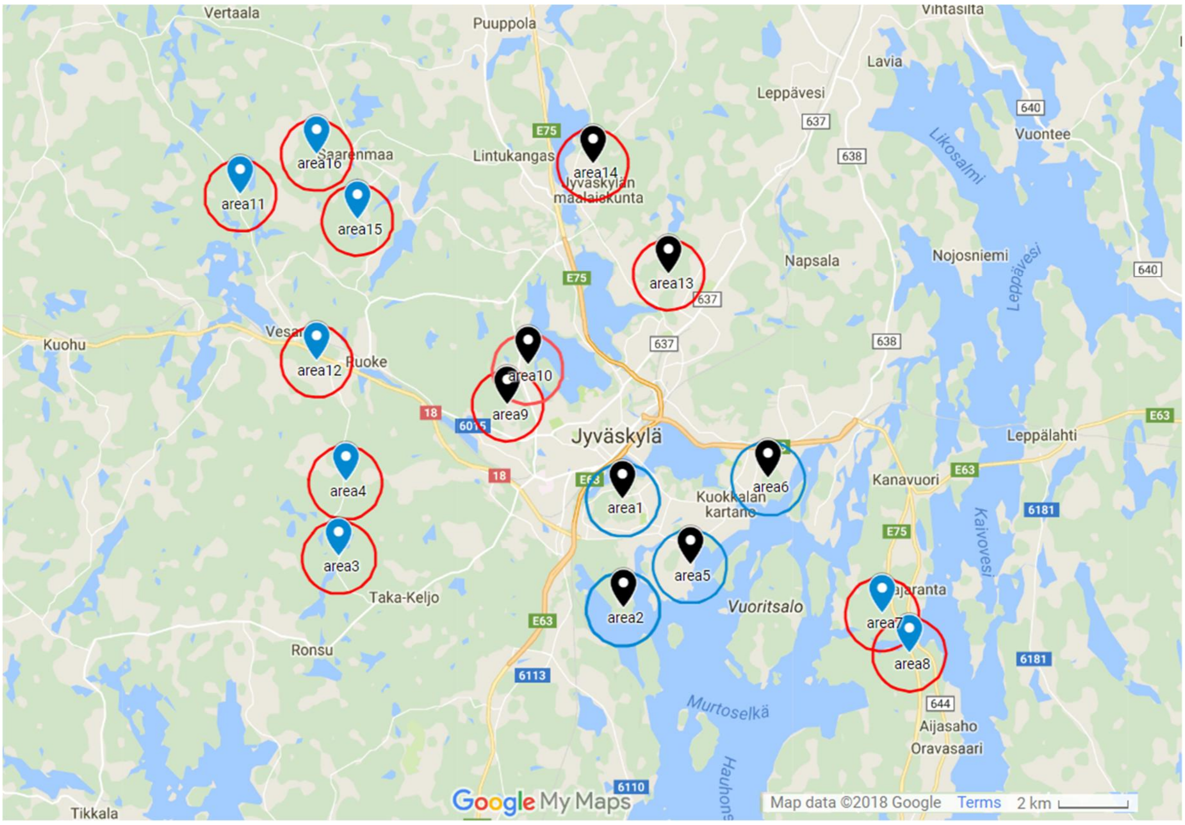

Figure S1. The location of the sampling areas (urban areas with black marks, non-urban areas with blue marks). Red lines delimits circular areas of 1 km radius centred on the sampling areas where *I. ricinus* was rare or absent. Blue lines delimits circular areas of 1 km radius centred on the sampling areas where *I. ricinus* was abundant. (Map data ©2018 Google; https:/google.com/maps, edited in Google My Maps).
